# A new dataset on milling time and public perception of Cell Broadcast tsunami alerts tested along the French Mediterranean coast on 19 January 2024

**DOI:** 10.1016/j.dib.2024.111073

**Published:** 2024-11-02

**Authors:** Johnny Douvinet, Fatima-Zahra Atmani, Maxime Deniaux, Matthieu Péroche, Noé Carles, Delphine Grancher

**Affiliations:** a Avignon University, UMR ESPACE 7300 CNRS, 74 rue Louis Pasteur, Case 19, F-84000 Avignon, France; b French University Institute, 1 rue Descartes, F-75231 05 PARIS Cedex, France; c Laboratoire de Géographie et Aménagement (LAGAM), route de Mende, F-34000, Montpellier, France; d Laboratoire de Géographie Physique (LGP), Thiais, Paris, 2 rue Henri Dunant, F-94320 Thiais, France

**Keywords:** Warning, Tsunami, Cell broadcast, Massive online questionnaire, France

## Abstract

Within the study of public perception and intended declarations in case of alert, an original dataset has been completed by using an online questionnaire, with a short URL link included in mobile alert messages, tested and displayed on 19 January 2024 along the French Mediterranean coast (engaging 189 municipalities and 9 departments). The aim is to further know and understand what people do and think upon receiving Cell Broadcast alerts, that deliver an attention-grabbing message directly on the screen of mobile phones of people located in the at-risk zones. A first notification was sent in the Tsunami Evacuation Zones from 09:30 to 10:30, and a second from 10:35 to 10:50 to close the test. A total of 9, 446 totally-completed answers have been collected during 2 days. The sample consists of 24 questions, designed by an interdisciplinary research team (including geographers, designers and psychologists researchers), to respond to a dual challenge: 1) firstly, to evaluate the participants’ immediate reactions to the Cell Broadcast messages, displayed with sound tone (that may provoke anxiety, fear or stress, particularly if individuals are confused by such type of alert), and 2) second, to estimate the intended milling time *(i.e.,* the time one person declared before he decides to evacuate) and to measure its influence for evacuation planning A few demographic details (age, professional status, location during alert reception) completed the dataset. The 9 Prefectures, the French Ministry of Interior and the researcher's team were involved before the test (to produce evacuation maps and alert messages), during the test (to locally observe reactions) and after the test (to present results to the practitioners). This original dataset serves as a critical resource for researchers, policymakers, and emergency managers focused on optimising Cell Broadcast alerts and defining alert messages. It is particularly suited to enhance the effectiveness and understanding of tsunami Cell Broadcast alerts.

Specifications Table

**Table:** 

Subject	Social Sciences: Geography; Psychology
Specific subject area	Evaluating what people do and think upon receiving a Cell-Broadcast emergency mobile alert, to contribute to producing new knowledge on public perception and intended declarations for crisis managers and emergency planning.
Type of data	Table (csv), Raw and filtered.
Data collection	The data was collected through an online questionnaire created by using Sphinx, a secured software. Respondents who received the Cell Broadcast alert message were invited to click on a short URL link, to answer 24 multiple-choice questions, addressing human behaviours and milling time. Notifications were broadcast on January 19, 2024, and data collection took place from January 19 to January 21, 2024. Respondents' consent was secured before participation.
Data source location	Institution: Avignon University City: Avignon Country: France.
Data accessibility	• Repository name: Mendeley • Data identification number: DOI: 10.17632/9wg3hb4w23.1 • Direct URL to data: https://data.mendeley.com/datasets/9wg3hb4w23/1
Related research article	Carles, N., Chapuis, K., Douvinet, J., Péroche, M. (2024). An Agent-Based Model to Simulate the Effects of Tsunami Warnings on Pedestrian Evacuation: Sensitivity Analysis and Early Findings, Proceedings of the 25th International Conference on Principles and Practice of Multi-Agent Systems, Kyoto (Japan), 18–24 nov. (accepted)

## 1 Value of the Data

• **Comprehensive Data on Public Perception on Content and Design of Alert Messaging:** This data allows the researchers to explore the degree of public perceptions of emergency alerts [1], offering a richer understanding of how people of various ages or professions react or act upon receiving alert messages with information Other questions focused on the drivers used in the *Warning Response Model* [2] and adapted during the last two decades to design clear and effective messages, to timely motivate recipients to apply protective actions [[3], [4], [5], [6]], WRM is the most accepted model as it reflects the desire to optimise messages in order to produce human decision-making [3]. This data allows researchers to measure if people well-perceive the WRM parameters, or if the latter needs to be adjusted.
• **Comprehensive Data on Milling Time:** The data provide detailed information on *milling time,* accepted as the time needed to prepare for evacuation [7,8]. Estimating the milling time is quite trickier since it involves complex psychological and sociological processes [7]. This time is currently estimated through local empirical surveys carried out in at-risk areas (as in the United States [9] or New Zealand [10], by asking individuals to project the time they would take before moving), or with surveys deployed post-event (such as the work carried out after the 2011 tsunami that occurred in Japan [11], the 2006 tsunami in Indonesia [12], the 2009 tsunami in Samoa [13] or the 2018 tsunami in the Sulawesi Islands in Indonesia [14].
• **Comparative Analysis with other surveys carried out with National Public Warning Systems referring to Cell Broadcast:** This dataset enables the comparisons with the public perception and milling time addressed with other Cell Broadcast systems, such as those existing in the United States, with WEA, Wireless Emergency Alerts [3]), in Japan, with the J-ALERT system [15], in the Netherlands with NL-Alert system [16] or in Austria [17]. By comparing the public perception, researchers can measure the influence of socio-cultural conditions, and improve emergency communication strategies in other cultural and technological contexts.
• **Enhancement of Evacuation and Crisis Management Models:** Researchers can use one part or the overall dataset to enhance crisis simulation and evacuation models. By incorporating more precise data on individuals' human behaviours and milling times, these models can be more accurate, leading to better planning and emergency planning. This, in turn, contributes to a more effective alert assessment and potentially saves lives during actual emergencies.
• **Cross-disciplinary Research Opportunities:** The interdisciplinary foundation—encompassing geography, psychology, design, and linguistics—makes it a valuable resource for researchers across various fields. It offers opportunities for cross-disciplinary studies, allowing experts from different domains to develop more holistic approaches to emergency management, particularly in understanding the human factors that influence response behaviours.
• **Baseline Data for Future Research:** This data can be reused by other researchers to track changes over time, assess the impact of technological advancements or policy changes, and explore trends in the public's response to emergency alerts, contributing to the ongoing improvement of emergency communication strategies.

## 2 Background

The French Ministry of Interior or the prefectures need to have information on the public perception and intended reactions to ensure that the Cell Broadcast alerts may be effective and really respond to the social needs in case of real alerts [6]. In this context, the French Ministry of Interior has commissioned our research team to conduct the study of public perception during trials tested since June 2022 in France with the Cell Broadcast solution and the so-called FR-Alert platform. Given the large variety of trials (*i.e.,* 190 trials from June 2022 to August 2024), the great diversity of scenarized dangers (*i.e.,* >12 different hazards or threats, such as industrial accidents, floods, fires) and the great number of answers collected (39, 974 answers during 75 trials), this study focused only on one exercise, addressed in the Tsunami Evacuation Zones (TEZ) along the French Mediterranean Sea. This dataset is expected to become the first contribution of public perception of CB in France (and in at large and massive scale as the CB zone equals to 201km2), and the emphasis on milling time was driven by its importance in emergency response research and the current lack of empirical data in this area, in comparison with other international datasets.

## 3 Data Description

The dataset has been structured into CSV files and supplementary files:

1. **CSV Files:**
  ○ **Tsunami_Test_Fr.csv:** This file contains the original survey data in French as it was collected from the participants. It includes all variables and responses in their original language.
  ○ **Tsunami_Test_En.csv:** This file is the English translation of the original dataset. It mirrors the structure and content of 1.csv but is translated for ease of use in an English-speaking context.
2. Supplementary Files:
  ○ **Data Dictionary:** This file provides a comprehensive list of all variable names, their descriptions, and the item values used in the dataset. It allows an understanding of the meaning and context of each variable and response.
  ○ **French-English Dictionary:** This file contains a Python dictionary of all the data, including variable names and response options, translated from French to English. It's the dictionary that was developed to translate the dataset, ensuring clarity and consistency between the original and translated datasets.
  ○ **Survey and survey translation:** This file has a table with all survey questions in French and their corresponding English translation.

These resources collectively ensure that both French and English-speaking users can engage with the dataset while maintaining consistency and transparency in the data translation. The survey aimed to assess public perception of mobile alerts, specifically those delivered via Cell Broadcast technology. Table 1 categorises the main variables, such as intended reactions to the alert, the perceived degree of danger, the “milling time”, and evacuation decisions, and the corresponding survey questions. Demographic variables (such as age, professional status and location during the alert diffusion) were also captured, along with media consumption habits during emergencies.

**Table 1:** Main topics and related variables embedded in the online questionnaire.

Survey topics	Collected variables and corresponding survey questions
*General Information*	(a) **N°Obs**: Observation identifier (Unique integer identifier)
*Consent and GDPR*	Q: Do you accept these conditions to start the survey? (a) **GDPR**: Consent to data collection
*Notification Reception*	Q: When did you receive the alert notification? (a) **notif_Date**: Date when the alert was received Q: When did you receive the alert notification? (a) **where_were_you:** Whereabouts when receiving the alert
*First Impressions of the Cell Broadcast Alerts*	Q: What was your first impression? What feelings were present right after the reception of a tsunami alert (a) **curiosity** (b) **misunderstanding** (c) **indifference** (d) **stress** (e) **surprise** (f) **fear**
*Response to Notification*	Q: After reading the notification, what did you do? (a) **erase:** Whether the alert notification was erased (b) **call:** Called someone to check if they received notification (c) **networks:** Checked social networks for more information (d) **prefecture:** Checked prefecture's website (e) **town hall:** Called the town hall (f) **help:** Called emergency services (g) **knew_how_to_react:** Knew the appropriate measures (h) **look_around**: Checked surroundings (i) **hesitate_to_act:** Hesitated to take action (j) **did_not_understand:** Did not comprehend actions expected
*Alert Notification Sound*	Q: If there was a sound associated with the notification, did you find that sound… (a) **pleasant_sound**: Was the sound pleasant? (b) **audible_sound**: Was the sound audible? (c) **intrusive_sound**: Was the sound intrusive (d) **stressful_sound**: Was the sound stressful? (e) **surprising_sound**: Was the sound surprising?
*The Alert Sender*	Q: According to you, the sender of the notification was… (a) **known_transmitter**: Was the sender recognized? (b) **credible_transmitter**: Was the sender credible? (c) **easy_transmitter**: Was the sender easy to recognize?
*Danger Description*	Q: The description of the danger was… (a) **understandable_danger**: Was the danger understandable? (b) **precise_danger**: Was the danger description precise? (c) **technical_danger**: Was the description too technical? (d) **complete_danger:** Was the description complete?
*Location of Event*	Q: The location of the event was… (a) **known_location**: Was the danger zone known? (b) **described_location**: Was the location clearly described? (c) **easy_location**: Was the location easy to locate?
*Instructions*	Q: The instructions were… (a) **understandable_instruction**: Were the instructions understandable? (b) **precise_instruction**: Were the instructions precise? (c) **long_instruction**: Were the instructions too long? (d) **useful_instruction**: Were the instructions useful?
*Notification Layout*	Q: The layout was… (a) **legible_Layout**: Was the layout legible? (b) **structured_Layout**: Was the layout well-structured? (c) **dense_Layout**: Was the layout dense?
*Notification Comprehension*	Q: Did you need to reread the notification? (a) **need_to_reread1**: Did the subject need to reread the notification?
*Demographic Information*	(a) **expected department**: The French department from which data was collected Q: What age group are you in? (b) **age_category**: Age category of the subject Q: Specify your profession and socio-professional category (c) **Socio-professional Category**: Subject's socio-professional category
*Evacuation Behavior*	Q: How long would it take you to evacuate? (a) **Evacuation_time**: The time the subject estimated for evacuation

**Fig. 1:**
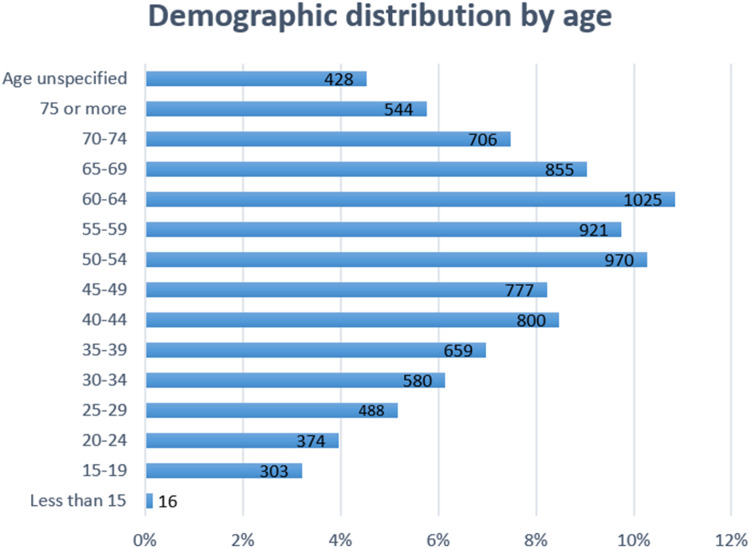
Demographic distribution of the respondents classified by age.

The respondents ranged in age from under 15 to over 75 years (Fig. 1). Compared to the 2024 INSEE population estimates, the sample exhibited few notable variations. Younger individuals (25–44) were overrepresented, particularly those aged 40–44 (8.5 %; +2.2 %) and 35–39 (7.0 %; +0.8 %). In contrast, older respondents were underrepresented, especially those aged 75 and above (5.8 %; −4.6 %). A greater proportion of respondents aged 60–64 participated (10.9 %, +4.7 %), and the 50–54 group also saw higher participation (10.3 %, +3.7 %). Meanwhile, younger groups, such as those aged 15–19 (3.2 %, −3.1 %) or 20–24 (4.0 %, −1.8 %), remain clearly underrepresented. A small percentage of respondents (4.5 %) did not specify their age [18].

**Fig. 2:**
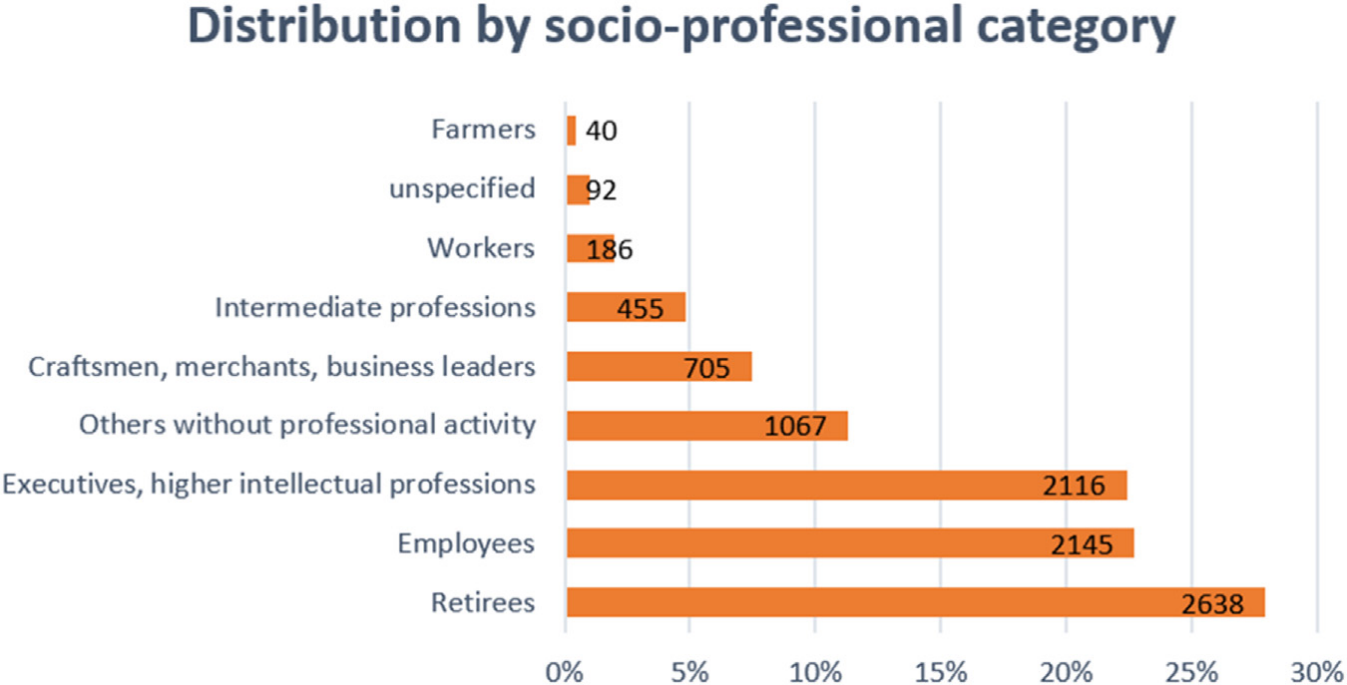
Distribution of the respondents classified by socio-professional category.

Furthermore, the socio-professional distribution of respondents shows deviations from the national averages provided by INSEE (Fig. 2). Retirees were well represented (27.93 %) compared to the national level, while the share of executives and higher intellectual professions was slightly higher (22.4 %, +0.8 %). However, intermediate professions were significantly underrepresented (4.82 %, −19.88 %), as workers (1.97 %, −17.13 %), compared to their respective national averages. Employees accounted for 22.73 % of respondents, a substantial underrepresentation compared to the national figure of 26.2 % (−3.47 %). Conversely, craftsmen, merchants, and business leaders were slightly more represented (7.46 %, +0.96 %), while farmers were notably fewer in this sample (0.42 %, −1.08 %). A small proportion of respondents (0.97 %, 92 individuals) did not specify their socio-professional category. These differences highlight the specific context of the sample, which has a much lower representation of intermediate professions and workers (because the test has been carried out during the morning of a Friday), whereas executives and retirees are more prominent. Despite these variations, the sample still provides useful insights into socio-professional distributions, though it may not fully reflect the broader population structure in France [19].

**Fig. 3:**
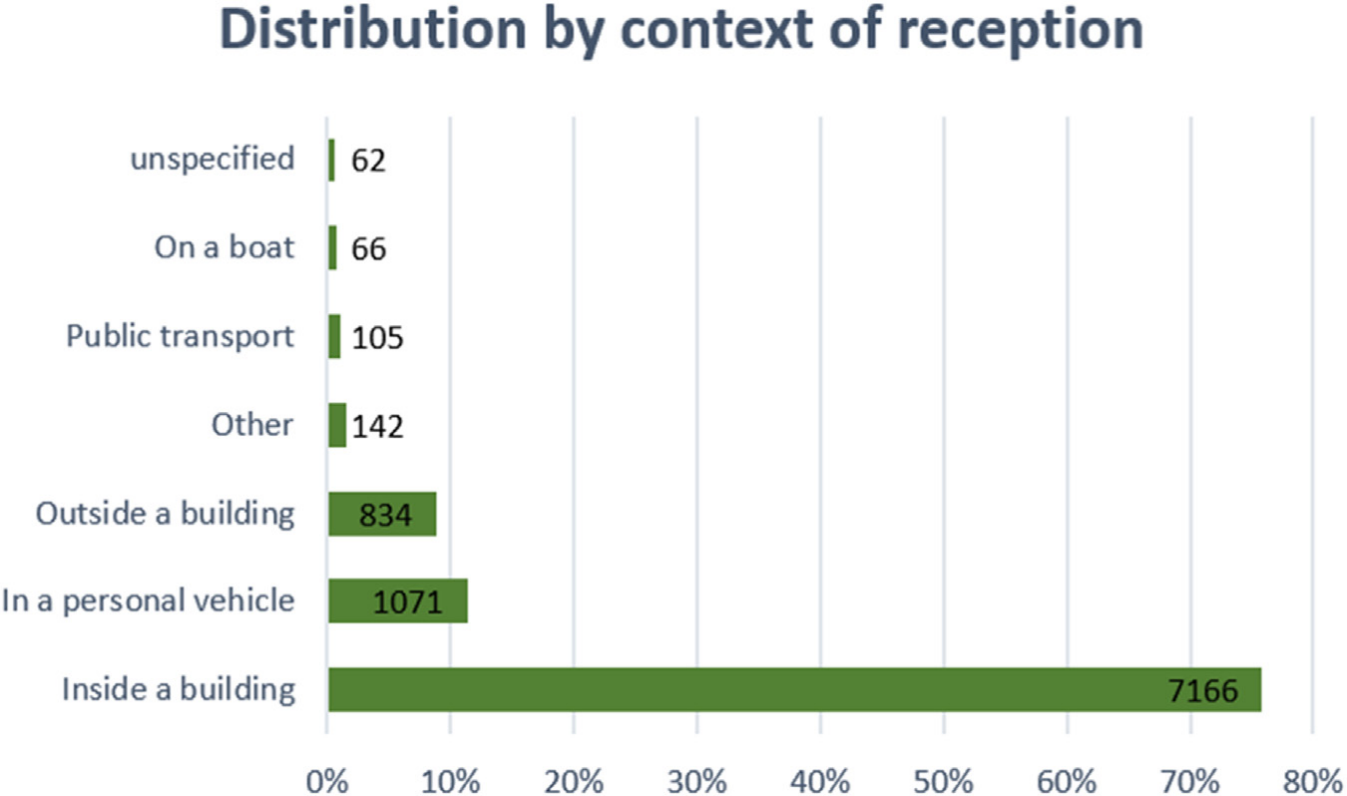
Distribution of the respondents classified by the reception context.

Among the 9, 446 respondents, the majority indicated that they were inside a building (75.9 %), while a smaller proportion reported being in a personal vehicle (11.3 %) or outside a building (8.8 %). Other less common locations included “other” (1.5 %), public transport (1.1 %), or on a boat (0.7 %). Only a small group (0.7 %) did not specify their location. The low proportion of outdoor participants could be explained by the fact that messages are less likely to be heard in urbanised areas, due to high levels of noise from traffic or environmental disturbances (Jagtman et al., 2010) (Fig. 3).

## 4 Experimental Design, Materials and Methods

**Fig. 4:**
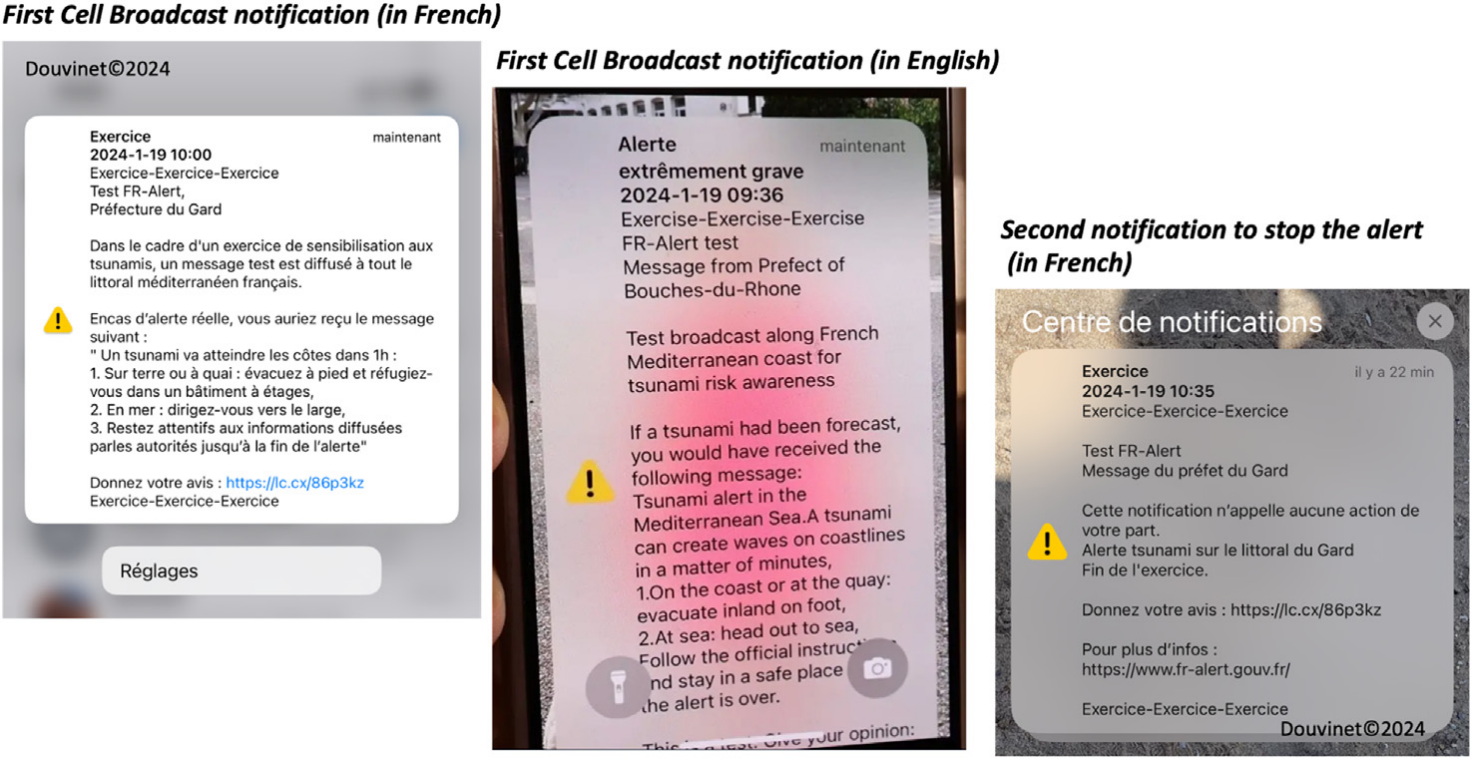
Screenshots of the different Cell Broadcast messages sent during the test.

**Data Collection Process:** The data was collected via a short URL link embedded in the Cell Broadcast alert message, diffused both in French and English language (Fig. 4). Data collection was managed through the Sphinx software that handled the distribution, survey and first analysis of answers during the trial and until the closure date of the survey. To answer the questionnaire, the recipients need to click on the short URL link (https://lc.cx/86p3kz), to limit the number of characters, but this link may be unknown, and considered spam. The recipients also have to look for the CB message on their phones if they have tried to cut off the CB tone during the reception.

**Collection Period:** The collection period takes place during two complete days, from January 19th at 09:30 until January 21st, 2024 at 12:00. 82.9 % ((*n* = 7, 825 answers) of the respondents declared they received the message between 09:30 and 10:50. 96.3 % of the respondents answer the questionnaire during the first day (*n* = 9, 107 answers).

**Fig. 5:**
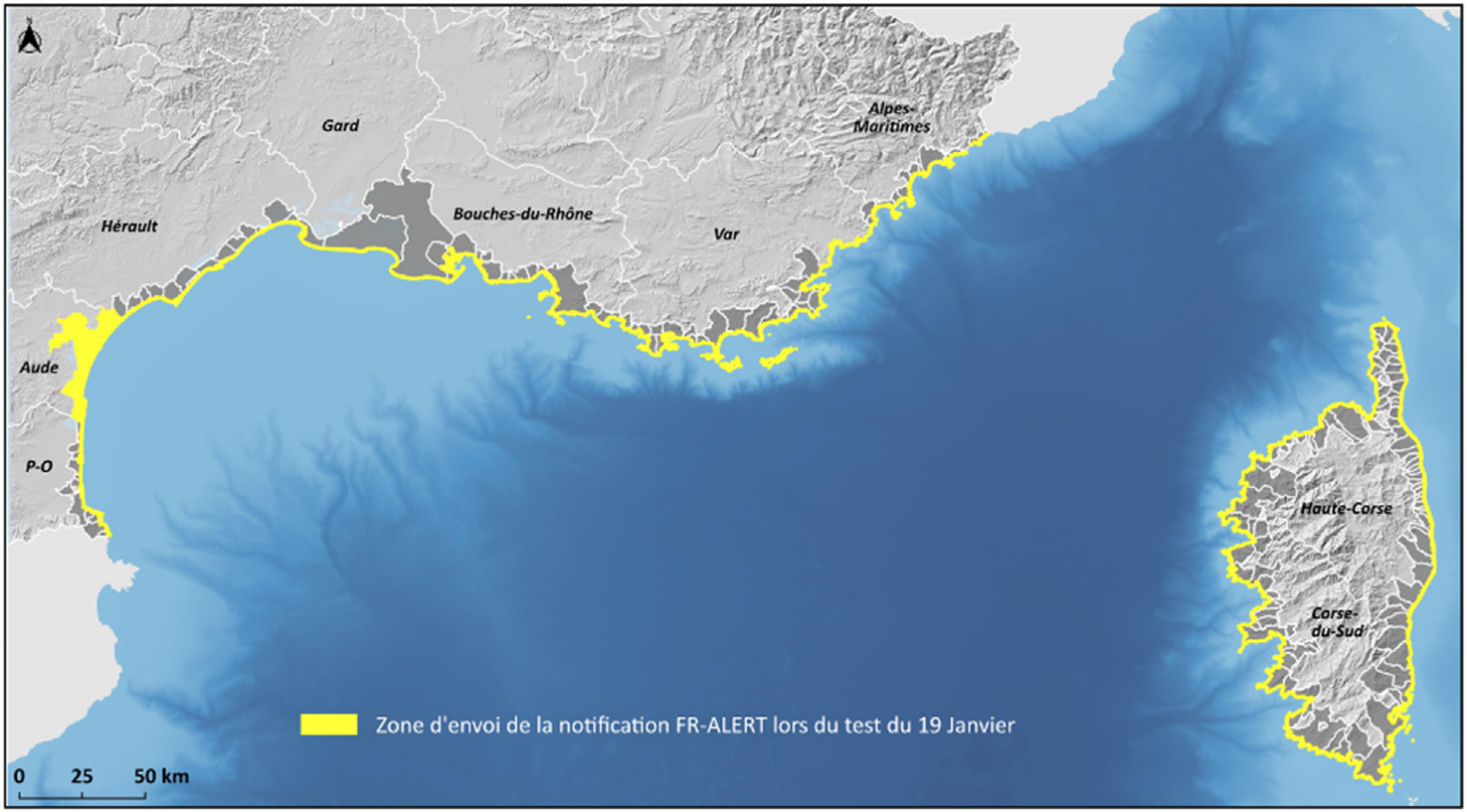
CB zone where the tsunami alert was sent during the trial on 19 January 2024.

**Data Collection Areas:** A cumulative area of 243 km2 has been covered by the CB alert, making the test of 19 January 2024 the one that broadcast an alert message over the largest spatial area ever covered in France and its overseas territories since June 2022. The Tsunami Evacuation Zones (TEZ) were used as the spatial reference to define the boundaries of the CB zone. The geometry of the zoning was too detailed to fit into the PAM, so a geometric simplification was carried out by removing a large number of vertices to reduce the size of the file (Fig. 5).

**Questionnaire Development:** The questionnaire was collaboratively designed by an interdisciplinary team of researchers specialising in geography, psychology, design, and linguistics. It consisted of 24 multiple-choice questions, with a focus on general reactions to the alert, six scientific invariants from existing literature, and demographic information such as age and socio-professional category. The questions were formulated to be understandable by individuals as young as 12 years old, ensuring broad accessibility and reliable data from a wide audience.

**Fig. 6:**
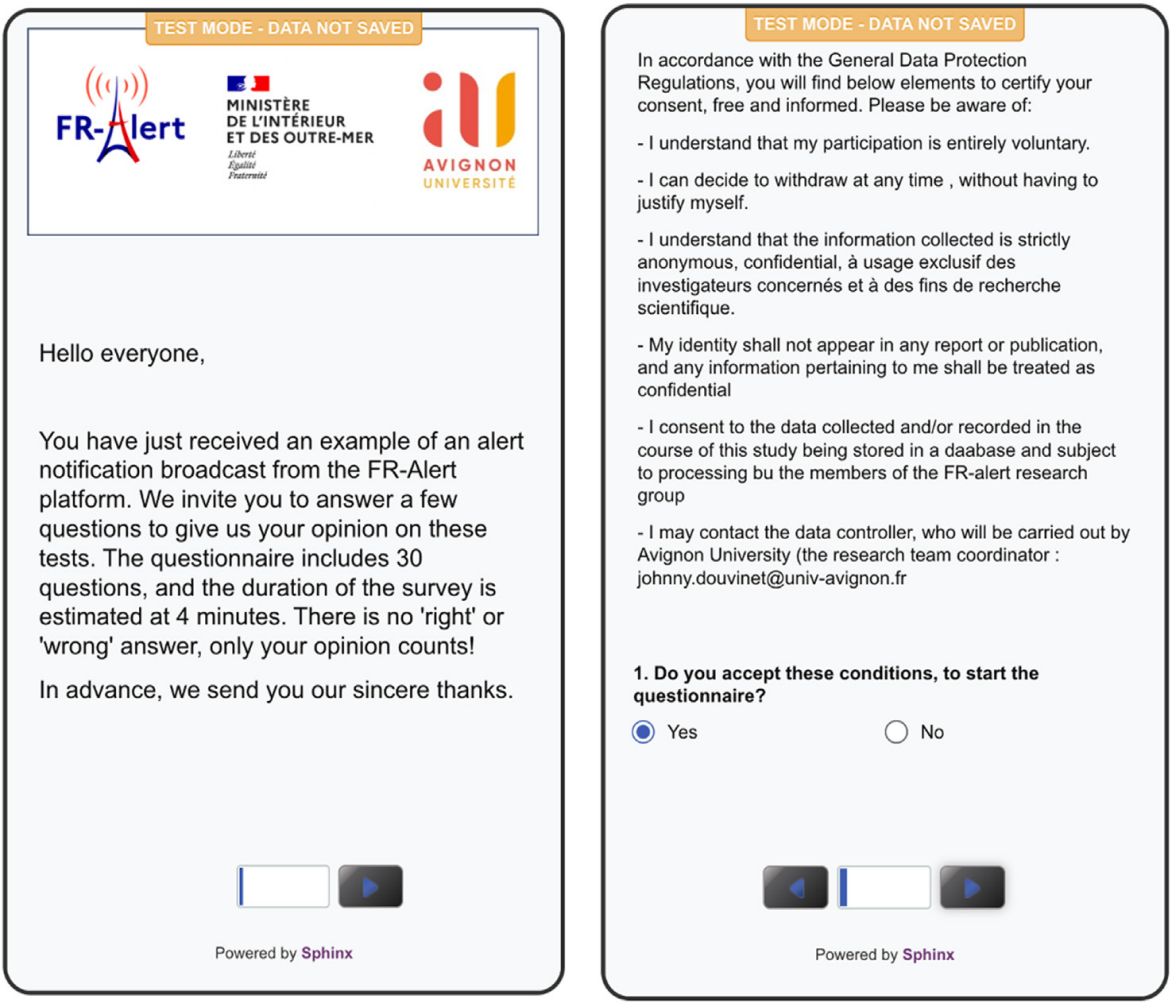
First and second pages of the questionnaire.

**Participant Consent:** Before starting the questionnaire, all the participants were required to provide informed consent. A concise and clear text summarising the sample usage conditions was presented on the first page. On the second page (Fig. 6), participants need to give their consent and agree to take part in the survey, respecting the European General Data Protection Regulations Directive, in application in France since 2015. Only those who accepted these terms proceed with the survey.

**Inclusion/Exclusion Criteria:** The dataset includes responses from individuals who received tsunami alert notifications and voluntarily agreed to complete the questionnaire.

**Translation Process:** To make the dataset accessible to a broader audience, the survey data was translated from French to English. This translation involved both manual and automated methods. A comprehensive French-English dictionary was manually created, covering all variable names and response values. Python scripts were then used to apply this dictionary to the entire dataset, ensuring that each value was accurately translated into English.

**Data Anonymization:** To protect participants' privacy and comply with GDPR, all identifying information was removed from the dataset. This included IP and MAC addresses, specific geolocations, and phone numbers. The anonymization process was rigorously applied to ensure that no personally identifiable information could be traced back to individual respondents.

**Data Cleaning:** Seven questions from the original survey were excluded from this dataset as they were relevant to scenarios other than the tsunami trial. Since this dataset focuses solely on the tsunami scenario, these questions were deemed irrelevant and were removed from the final dataset. Thus, this dataset finally includes the answers to 17 out of the 24 questions. The exclusion ensured the dataset's specificity and its completeness. Furthermore, the final data collection includes 9, 446 answers, but 315 responses (3.3 % of the initial data collection) have been deleted when >4 questions were uncompleted.

## Limitations

**Socio-Professional Bias:** This data collection process had several limitations: 1) people had to click on the unknown URL link to answer the questionnaire; 2) they have to trust in it, 3) they have to well receive messages during this trial; 4) they have to know how to retrieve the notifications on their own phones. On the other hand, the data represents a great and large variety of socio-professional categories, surely with an overrepresentation of executives, older persons or service executives, and an underrepresentation of workers, who typically have less time to respond to online surveys.

**Under-Representation of Younger Population:** The survey under-represented individuals under 15 years old, as such data collection requires the consent and agreement of parents.

## Ethics Statement

All the authors have read and followed the ethical requirements for publication in Data in Brief and confirmed that the current work does not involve animal experiments, or any data collected from AI or social media platforms. Our work involved human subjects and was carried out after obtaining informed consent from all participants before data collection, in compliance with GDPR (Fig. 6). Additionally, the dataset eliminated all identifying information; IP and MAC addresses, specific geolocations, and phone numbers.

## Credit Author Statement

**Johnny Douvinet:** Project administration, Supervision, Sample supervision, writing draft, Limitations, Background & Design experimentations; **Fatima-Zahra Atmani:** Data collection, writing draft & draft preparation; **Maxime Deniaux:** Data curation; **Delphine Grancher:** Sample Supervision, Reviewing; **Noé Carles**: Map of the CB zone; **Matthieu Péroche**: Design of the tsunami alert message, map of the Tsunami Evacuation Zones (TEZ).

## Data Availability

Mendeley DataMilling time and public perception of Cell Broadcast tsunami alerts tested on the French Mediterranean coast on 19 January 2024 (Original data).

## Appendix Supplementary materials

Application 1

Application 2

Application 3
