# Supplementary material for: A new dataset on milling time and public perception of Cell Broadcast tsunami alerts tested along the French Mediterranean coast on 19 January 2024
Source: Data Brief. 2024 Nov 2;57:111073. doi: 10.1016/j.dib.2024.111073 (PMC11599998; doi:10.1016/j.dib.2024.111073)
Supplement: Supplementary file 1 [file mmc1.docx]

Key information:

Variable name

Variable description

Item values

*All “Nan” values represent instances where the data subject preferred not to disclose the information

| Variable | Description | Value |
| --- | --- | --- |
| N°Obs | Observation identifier | Unique Integer identifier |
| Q: Do you accept these conditions to start the survey? | | |
| GDPR | If the data subject consented to their data being collected and used | Yes  **Though the survey had a yes and a no choice to this question, the dataset only contains a yes value, as it was necessary to give informed consent to start the survey* |
| Q: When did you receive the alert notification? | | |
| notif_Date | The date when the data subject received the alert notification | 19/01/2024  20/01/2024  21/01/2024 |
| Q: Where were you when you received the Alert notification? | | |
| where_were_you | The whereabouts of the data subject at the time of the alert | Inside a building  In a personal vehicle  Outside a building  Public transport  On a boat  Other  *Nan* |
| Q: What was your first impression? | | |
| curiosity | What feelings were present right after the reception of a tsunami alert | Yes  No  I don't remember  *Nan* |
| misunderstanding |  |  |
| indifference |  |  |
| stress |  |  |
| surprise |  |  |
| fear |  |  |
| Q: After reading the notification, what did you do? | | |
| erase | The data subject erased the notification | Yes  No  I don't remember  *Nan* |
| call | Called someone to check if they had received a notification |  |
| networks | Checked social networks for more information |  |
| prefecture | Checked the prefecture’s website |  |
| town hall | Called the town hall |  |
| help | Called the emergency number |  |
| knew_how_to_react | Knew the appropriate measures to take |  |
| look_around | Checked surroundings |  |
| hesitate_to_act | Hesitated to take action |  |
| did_not_understand | Did not comprehend what actions were expected |  |
| Q: If there was a sound associated with the notification, did you find that sound... | | |
| pleasant_sound | The impression the notification sound left on the data subject | Yes  No  I don't remember  *Nan* |
| audible_sound |  |  |
| intrusive_sound |  |  |
| stressful_sound |  |  |
| surprising_sound |  |  |
| Q: According to you, the sender of the notification was... | | |
| known_transmitter |  | Yes  No  I don't remember  *Nan* |
| credible_transmitter |  |  |
| easy_transmitter |  |  |
| Q: The description of the danger was... | | |
| understandable_danger | The alert described the danger in an understandable way | Yes  No  I don't remember  *Nan* |
| precise_danger | The alert described the danger in a precise way |  |
| technical_danger | The description was too technical |  |
| complete_danger | The description was complete and did not lack important information |  |
| Q: The location of the event was… | | |
| known_location | The danger zone was specified in the notification | Yes  No  I don't remember  *Nan* |
| described_location | The danger zone was clearly described |  |
| easy_location | The danger zone was easy to locate |  |
| Q: The instructions were... | | |
| understandable_instruction | The alert included understandable instructions | Yes  No  I don't remember  *Nan* |
| precise_instruction | The alert included precise instructions |  |
| long_instruction | The instructions in the alert were too long |  |
| useful instruction | The instructions in the alert were useful |  |
| Q: The layout was... | | |
| legible_Layout | The alert notification’s layout was legible | Yes  No  I don't remember  *Nan* |
| structured_Layout | The alert notification’s layout was well-structured |  |
| dense_Layout | The alert notification’s layout was dense |  |
| Q: Did you need to reread the notification? | | |
| need_to_reread1 | It was necessary to read the notification multiple times to understand it | Yes  No |
|  | | |
| expected department | The French department from which the data was collected | 06-Alpes-Maritimes  11-Aude  13-Bouches-du-Rhone  2A-Corse-du-Sud  2B-Haute-Corse  30-Gard  34-Herault  66-Pyrenees-Orientales  83-Var |
|  | | |
| scenario | The scenario the Alert was simulating | tsunami |
| Q: What age group are you in? | | |
| age_category | The age category the data subject falls within | Less than 15  15-19  20-24  25-29  30-34  35-39  40-44  45-49  50-54  55-59  60-64  65-69  70-74  75 or more  *Nan* |
| Q: Specify your profession and socio-professional category | | |
| Socio-professional Category | The social category the data subject falls within | Retirees  Employees  Executives, higher intellectual professions  Others without professional activity  Craftsmen, merchants, business leaders  Intermediate professions  Workers  Farmers  *Nan* |
| Q: How long would it take you to evacuate? | | |
| Evacuation_time | The data subjects' estimation of the evacuation time | less than 1 minute  2 to 5 minutes  6 to 10 minutes  11 to 20 minutes  21 to 30 minutes  more than 31 minutes  I do not know  I would not have evacuated so I will not answer this question  Question not asked  *Nan* |
