# Supplementary material for: A new dataset on milling time and public perception of Cell Broadcast tsunami alerts tested along the French Mediterranean coast on 19 January 2024
Source: Data Brief. 2024 Nov 2;57:111073. doi: 10.1016/j.dib.2024.111073 (PMC11599998; doi:10.1016/j.dib.2024.111073)
Supplement: Supplementary file 3 [file mmc3.docx]

Survey and Survey translation

| Originale (French) | English |
| --- | --- |
| Bonjour à toutes et à tous,    Vous venez de recevoir un exemple de notification d'alerte diffusée depuis la plateforme FR-Alert. Nous vous invitons à répondre à quelques questions, pour nous donner votre avis sur ces tests. Le questionnaire comprend 18 questions, et la durée de l'enquête est estimée à 4 minutes. Il n'y a pas de "bonne" ou de "mauvaise" réponse, seul votre avis compte !  Par avance, nous vous adressons nos sincères remerciements. | Hello everyone,  You have just received an example of an alert notification broadcast from the FR-Alert platform. We invite you to answer a few questions to give us your opinion on these tests. The questionnaire includes 18 questions, and the duration of the survey is estimated at 4 minutes. There is no “right” or “wrong” answer, only your opinion counts!  In advance, we send you our sincere thanks. |
| En accord avec le Règlement Général sur la Protection des Données, vous trouverez ci-dessous des éléments pour certifier votre consentement, libre et éclairé. Merci d’en prendre connaissance :  - Je comprends que ma participation est entièrement volontaire.  - Je peux décider de me retirer à tout moment, sans avoir à me justifier.  - Je comprends que les informations récoltées sont strictement anonymes, confidentielles, à usage exclusif des investigateurs concernés et à des fins de recherche scientifique.  - Mon identité n’apparaîtra dans aucun rapport ou publication et toute information me concernant sera traitée de façon confidentielle.  - J’accepte que les données recueillies et/ou enregistrées à l’occasion de cette étude puissent être conservées dans une base de données et faire l’objet d’un traitement par les membres du groupe de recherche FR-Alert  Je peux contacter le responsable du traitement qui sera réalisé par Avignon Université (coordinateur de l’équipe de recherche) : johnny.douvinet@univ-avignon.fr **Acceptez-vous ces conditions, pour commencer le questionnaire ?**  - Oui - Non | In accordance with the General Data Protection Regulations, you will find below elements to certify your free and informed consent. Please read it:  - I understand that my participation is entirely voluntary.  - I can decide to withdraw at any time, without having to justify myself.  - I understand that the information collected is strictly anonymous, confidential, for the exclusive use of the investigators concerned and for scientific research purposes.  - My identity will not appear in any report or publication and any information concerning me will be treated confidentially.  - I accept that the data collected and/or recorded during this study may be stored in a database and processed by members of the FR-Alert research group  I can contact the person responsible for the processing which will be carried out by Avignon University (coordinator of the research team): johnny.douvinet@univ-avignon.fr  **Do you accept these conditions to begin the questionnaire?**   - Yes - No |
| **Quand avez-vous reçu la notification d'alerte ?****A quelle heure avez vous reçu la notification d'alerte ?** | **When did you receive the alert notification?**  **What time did you receive the alert notification?** |
| **Dans quel pays étiez-vous?**   - France - Andorre - Allemagne - Belgique - Espagne - Italie - Luxembourg - Monaco - Suisse | **What country were you in?**   - France - Andorra - Germany - Belgium - Spain - Italy - Luxembourg - Monaco - Swiss |
| **Peut-être pouvez-vous nous donner le nom d'une commune proche de là où vous étiez?** | **Perhaps you can give us the name of a town close to where you were?** |
| **Où étiez-vous au moment de la réception de la notification ?**  - A l'extérieur (en plein air, rue, parc, à pied , en vélo, en trotinette...) - A l'intérieur d'un bâtiment (chez moi, dans un magasin, au travail, à l 'école , chez le médecin...) - Autre - En transports en commun, bus , train - En voiture, moto, camion - Sur un bateau | **Where were you when you received the notification?**   - Outside (outdoors, street, park, on foot, by bike, scooter, etc.) - Inside a building (at home, in a store, at work, at school, at the doctor's office, etc.) - Other - By public transport, bus, train - By car, motorcycle, truck - On a boat |
| **Quelle était la nature du danger simulé ?**  - Risque cyclonique - Risque tsunami - Risque séisme - Risque feu de forêt - Risque industriel - Risque nucléaire - Risque inondation - Risque attentat - Risque rupture de barrage - Je ne sais plus - Autre | **What was the nature of the simulated danger?**   - Cyclone risk - Tsunami risk - Earthquake risk - Forest fire risk - Industrial risk - Nuclear risk - Flood risk - Risk of attack - Risk of dam failure - I don't know anymore - Other |
| **Quelle a été votre première impression ?** Pour chaque sentiment ci-dessous, répondez par "oui", "non", ou "je ne sais plus".   1. L'agacement 2. La curiosité : 3. L'incompréhension : 4. L'indifférence : 5. Le stress : 6. La surprise : 7. La peur : | **What was your first impression?**  For each feeling below, answer “yes,” “no,” or “I don’t know.”   1. Annoyance 2. Curiosity: 3. Misunderstanding: 4. Indifference: 5. Stress: 6. Surprise: 7. Fear: |
| **Après lecture de la notification, qu'avez-vous fait ?** Pour chaque action ci-dessous, répondez par "oui", "non", ou "je ne sais plus".   1. J'ai effacé la notification : ___ 2. J'ai appelé quelqu'un pour lui demander s'il avait reçu la notification : ___ 3. J'ai consulté les réseaux sociaux : ___ 4. J'ai consulté le site de la préfecture : ___ 5. J'ai appelé les secours : ___ 6. J'ai su comment réagir : ___ 7. J'ai regardé autour de moi : ___ 8. J'ai hésité à agir : ___ 9. Je n'ai pas compris ce qu'il fallait faire : ___ | **After reading the notification, what did you do?** For each action below, respond with "yes," "no," or "I don't know."   1. I deleted the notification: ___ 2. I called someone to ask if they had received the notification: ___ 3. I checked social media: ___ 4. I visited the prefecture's website: ___ 5. I called emergency services: ___ 6. I knew how to react: ___ 7. I looked around me: ___ 8. I hesitated to act: ___ 9. I did not understand what to do: ___ |
| **En cas d'alerte réelle,** Pour chaque situation ci-dessous, répondez par "pas du tout", "peut-être", ou "sûrement".   1. Je me serais mis(e) à l'abri : ___ 2. Je me serais éloigné(e) du danger : ___ 3. J'aurais appelé les secours : ___ 4. Je serais allé(e) chercher mes enfants à l'école : ___ 5. J'aurais poursuivi mes activités : ___ 6. J'aurais appliqué les consignes : ___ 7. Je ne sais pas ce que j'aurais fait : ___ | **In case of a real alert,** For each situation below, respond with "not at all", "maybe", or "definitely".   1. I would have taken shelter: ___ 2. I would have moved away from danger: ___ 3. I would have called emergency services: ___ 4. I would have gone to pick up my children from school: ___ 5. I would have continued my activities: ___ 6. I would have followed the instructions: ___ 7. I don't know what I would have done: ___ |
| **Si un son était associé à la notification, avez-vous trouvé ce son...** Pour chaque adjectif ci-dessous, répondez par "oui", "non", ou "je ne sais plus".   1. Agréable : ___ 2. Audible : ___ 3. Intrusif : ___ 4. Stressant : ___ 5. Surprenant : ___ | **If a sound was associated with the notification, did you find the sound...** For each adjective below, respond with "yes", "no", or "I don't remember".   1. Pleasant: ___ 2. Audible: ___ 3. Intrusive: ___ 4. Stressful: ___ 5. Surprising: ___ |
| **D'après vous, l'émetteur de la notification était...**  Pour chaque question ci-dessous, répondez par "oui", "non", ou "je ne sais plus".   1. Connu : ___ 2. Crédible : ___ 3. Facile à identifier : ___ | **In your opinion, was the sender of the notification...**  For each question below, respond with "yes", "no", or "I don't remember".   1. Known: ___ 2. Credible: ___ 3. Easy to identify: ___ |
| **La description du danger était...** Pour chaque question ci-dessous, répondez par "oui", "non", ou "je ne sais plus".   1. compréhensible: ___ 2. précise: ___ 3. trop technique: ___ 4. complète: ___ | **The description of the danger was...**  For each question below, answer “yes”, “no”, or “I don’t know”.   1. understandable: ___ 2. precise: ___ 3. too technical: ___ 4. complete: ___ |
| **La localisation de l'évènement était :** Pour chaque question ci-dessous, répondez par "oui", "non", ou "je ne sais plus".   1. Connue : ___ 2. Clairement décrite : ___ 3. Facile à localiser : ___ | **The location of the event was:** For each question below, respond with "yes", "no", or "I don't remember".   1. Known: ___ 2. Clearly described: ___ 3. Easy to locate: ___ |
| **Les consignes étaient...** Pour chaque question ci-dessous, répondez par "oui", "non", ou "je ne sais plus".   1. Compréhensibles : ___ 2. Précises : ___ 3. Trop longues : ___ 4. Utiles : ___ 5. Complètes : ___ 6. Adaptées à la situation : ___ | **The instructions were...** For each question below, respond with "yes", "no", or "I don't remember".   1. Understandable: ___ 2. Precise: ___ 3. Too long: ___ 4. Useful: ___ 5. Complete: ___ 6. Appropriate for the situation: ___ |
| **La mise en page était...** Pour chaque question ci-dessous, répondez par "oui", "non", ou "je ne sais plus".   1. Lisible : ___ 2. Structurée : ___ 3. Dense : ___ | **The layout was...** For each question below, respond with "yes", "no", or "I don't remember".   1. Readable: ___ 2. Structured: ___ 3. Dense: ___ |
| **Avez-vous eu besoin de relire la notification ?**   - Oui - Non   **Pourquoi avez-vous eu besoin de relire la notification ?**   - Pour comprendre ce qu'il se passait - Pour la montrer autour de moi - Pour répondre à ce questionnaire - Avez-vous essayé de couper le son ? - Oui - Non | **Did you need to reread the notification?**   - Yes - No   **Why did you need to reread the notification?**   - To understand what was happening - To show it to others - To answer this questionnaire - Did you try to mute the sound? - Yes - No |
| **Quel est votre genre ?**   - Féminin - Masculin - Je ne souhaite pas répondre - Autre   **Dans quelle tranche d'âge êtes-vous ?**   - 15-19 - 20-24 - 25-29 - 30-34 - 35-39 - 40-44 - 45-49 - 50-54 - 55-59 - 60-64 - 65-69 - 70-74 - 75 ans ou plus - Moins de 15 ans - Non réponse | **What is your gender?**   - Female - Male - Prefer not to answer - Other   **What is your age range?**   - 15-19 - 20-24 - 25-29 - 30-34 - 35-39 - 40-44 - 45-49 - 50-54 - 55-59 - 60-64 - 65-69 - 70-74 - 75 or older - Under 15 - No response |
| **Précisez votre profession et catégorie socio-professionnelle :**   - Agriculteurs exploitants - Artisans, commerçants, chefs d'entrepriseCadres, professions intellectuelles supérieures - Professions intermédiaires - Employés - Ouvriers - Retraités - Autres sans activité professionnelle | **Specify your profession and socio-professional category:**   - Farmers - Craftsmen, merchants, business leaders - Executives, higher intellectual professions - Intermediate professions - Employees - Workers - Retirees - Others without professional activity |
| **Sur quel(s) appareil(s) avez-vous reçu la notification ?**   - Smartphone Android - Smartphone iOS (Apple) - Tablette - Voiture - Autre (montre connectée, etc.)   **Votre téléphone est-il habituellement en mode silencieux ou vibreur ?**   - Oui - Non | **Which device(s) did you receive the notification on?**   - Android smartphone - iOS (Apple) smartphone - Tablet - Car - Other (smartwatch, etc.)   **Is your phone usually in silent or vibrate mode?**   - Yes - No |
| **Pour finir, n'hésitez pas à nous laisser un commentaire !** | **Finally, don’t hesitate to leave us a comment!** |
